# Key Aspects in Designing High-Throughput Workflows in Electrocatalysis Research: A Case Study on IrCo Mixed-Metal Oxides

**DOI:** 10.1021/acsmaterialslett.4c01372

**Published:** 2024-10-15

**Authors:** Joanna M. Przybysz, Ken J. Jenewein, Mária Minichová, Tomáš Hrbek, Thomas Böhm, Tatiana Priamushko, Serhiy Cherevko

**Affiliations:** † Helmholtz-Institute Erlangen-Nürnberg for Renewable Energy (IET-2), Forschungszentrum Jülich, Cauerstrasse 1, 91058 Erlangen, Germany; ‡ Department of Chemical and Biological Engineering, Friedrich-Alexander-Universität Erlangen-Nürnberg, Egerlandstrasse 3, 91058 Erlangen, Germany; § Charles University, Faculty of Mathematics and Physics, Department of Surface and Plasma Science, V Holešovičkách 2, 180 00 Prague 8, Czech Republic

## Abstract

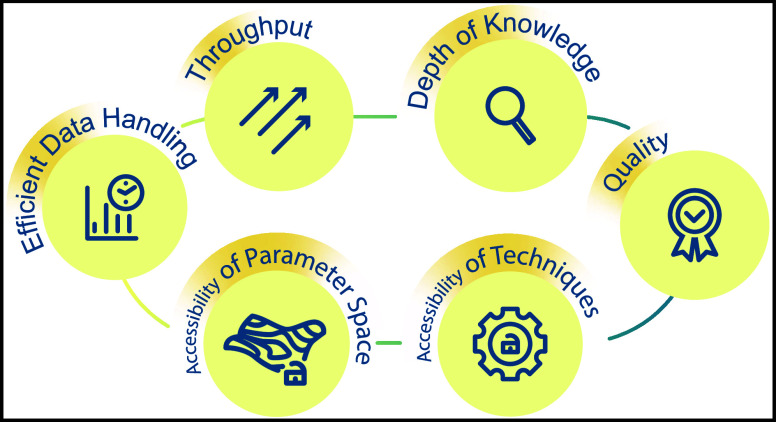

With the growing interest of the electrochemical community in high-throughput (HT) experimentation as a powerful tool in accelerating materials discovery, the implementation of HT methodologies and the design of HT workflows has gained traction. We identify 6 aspects essential to HT workflow design in electrochemistry and beyond to ease the incorporation of HT methods in the community’s research and to assist in their improvement. We study IrCo mixed-metal oxides (MMOs) for the oxygen evolution reaction (OER) in acidic media using the mentioned aspects to provide a practical example of possible workflow design pitfalls and strategies to counteract them.

The recent rise of interest in high-throughput (HT) experimentation and self-driving laboratories (SDLs) in materials science and electrocatalysis arises from the burning need for technological advancements to tackle humanity’s problems, such as climate change and resource limitations.^1,2^ The acceleration of research efforts brought by automated and autonomous methods is tempting to shorten the time-to-market for new materials. Such endeavors result in a reformation of research further driven by projects such as the Materials Genome Initiative.^3^

The discovery of materials has so far mostly relied on serendipity, rather than systematic large-scale testing of materials. This is not surprising, considering the number of parameters that affect material performance and the multiobjective nature of the search for the best candidate. Nowadays, technology is advanced enough to offer alternatives to conventional, inefficient research efforts through automation and parallelization of tasks that constitute the workflow (HT experimentation), and even shifting from the classical design of experiment (DoE) approaches to artificial intelligence (AI)-driven research campaigns.^4,5^ While HT approaches are generally designed to screen large amounts of samples, they also accelerate studies involving smaller sample numbers. It should be noted that DoE strategies can be incorporated into HT experimental design to further increase research efficiency.^6^ AI-guided experimentation offers an alternative experimental design approach, potentially further increasing efficiency and bridging the gap between HT screening (limited property extraction) and slow fundamental research (rich property extraction), especially when combined with coupled characterization techniques.^7^

An HT workflow commonly consists of combinatorial synthesis, rapid characterization, and automated data analysis.^8,9^ We believe that the building blocks for HT campaigns are already available, and at this point, it is possible to pick and assemble workflows according to the chosen research objectives. It is, however, essential to spark discussion on the best practices in HT workflow development. Therefore, we identify a need to provide guidelines specific to HT electrocatalyst research. This is essential to produce reliable data of high value and reusability, which is of specific importance for research involving AI algorithms feeding on literature data, as emphasized by the FAIR (Findable, Accessible, Interoperable, Reusable) principles for scientific data.^10^

Here, we identify important aspects for the design of HT electrocatalysis research workflows, building and expanding upon ideas previously reported in the literature. The mentioned aspects aim to aid researchers looking to implement HT approaches in their work, as well as bring attention to potential pitfalls of flawed HT workflow design. We run an exemplary case study on the activity and stability of IrCo mixed metal oxides (MMOs) to provide a practical demonstration of several of those aspects.

The choice of the model system was motivated by the catalyst-related challenges faced by the proton exchange membrane water electrolysis (PEMWE) technology used for green hydrogen production. In this technology, catalysts must be able to withstand highly acidic and oxidizing environment to support long-term electrolyzer operation. Up to now, only IrO_2_ meets this requirement, but Ir is expensive and scarce.^11^ Reducing the loading of Ir-based catalysts used to drive the anodic oxygen evolution reaction (OER) is a critical objective since material scarcity constitutes a bottleneck for the technology scale-up.^12−14^

Partial substitution of Ir with non-noble, OER-active metals is one strategy, offering the possibility to tune the electronic properties of the Ir catalyst and further enhance its OER activity.^15,16^ Numerous substitute candidates have been proposed, many of which, however, are thermodynamically unstable in the operating conditions, as demonstrated by Pourbaix diagrams.^15,17^ This limitation, however, is not definite since element stability in mixed phases may differ, and slow dissolution kinetics may shift the scale in favor of overall material stability.^18^ As an exemplary system, we choose IrCo MMOs, as Co oxides were reported to have a high OER activity in acid and moderate stability.^15,19−21^

HT approaches are well suited to conduct the search for active and stable MMOs. However, several aspects of the experimental design must be addressed to ensure representative screening and high-quality experimental findings. Within this work, we highlight aspects through which we evaluate our model HT workflow design and encourage other HT practitioners in the field of electrocatalysis to consider too. We identify a total of 6 aspects, including Throughput, Depth of Knowledge, Quality, Accessibility of Techniques, Accessibility of Parameter Space, and Efficient Data Handling, summarized in Table 1. The table also includes an evaluation of the model study presented in this work, in terms of the 6 aspects, with an assigned grade on a five-point scale concerning each aspect. The subjective rating is granted by the researcher. It is presented here to illustrate workflow evaluation. A higher grade signifies a higher degree of satisfaction of a given aspect by the model workflow (Table 1).

**Table 1:**
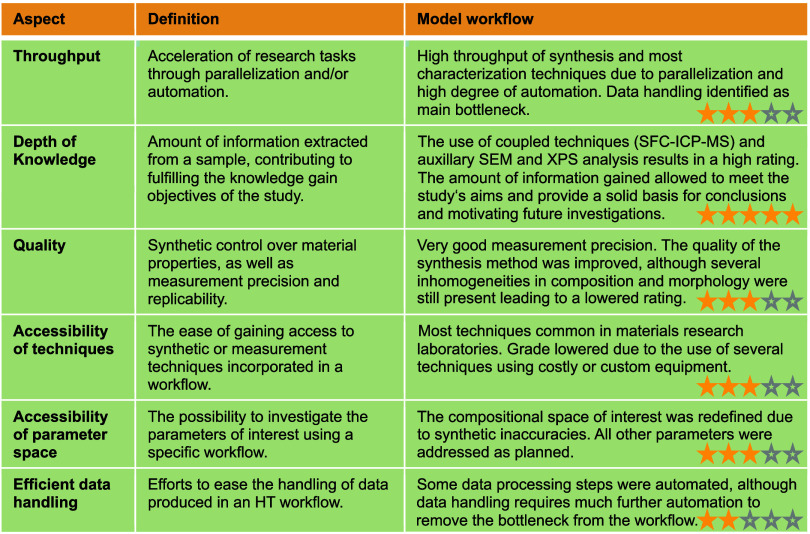
A Summary of the 6 Aspects for HT Workflow Design and the Evaluation of the Model Workflow for IrCo MMOs Presented in This Study^a^

a A rating on a 5-point scale represented by star icons corresponds to the degree of satisfaction of a given aspect by the model workflow.

## Throughput

HT workflows are primarily characterized by the acceleration of experiments, which can relate to the achieved increase in throughput in a given campaign or the maximum achievable throughput estimated for the platform. The throughput is often dependent on the research objectives. For example, a platform that is designed for electrochemical degradation testing will, by default, be slower than those only targeting electrocatalytic activity using less time-intensive protocols. In principle, parallelization of long-term experiments can increase the experimental throughput of such systems, although it may lead to a large increase in the total costs.

## Depth of Knowledge (Sample Exploitation)

This aspect describes the amount of complementary information that can be extracted from one sample. Sample exploitation correlates to the amount of knowledge that can be gained about the studied system within one campaign. Thorough characterization of samples with multiple methods adds to the total amount of knowledge about the studied system.

One strategy to maintain the throughput while expanding the depth of knowledge from one experiment is using multimodal *in situ* and *operando* analytical techniques, which allow for simultaneous measurements of various parameters and provide information on the processes occurring during catalyst operation that are otherwise unobtainable. The use of this strategy, however, depends on the research objectives. A wide range of spectroscopic techniques has been reported up to date, including in situ IR and Raman spectroscopy,^22,23^ (surface) X-ray diffraction ((S)XRD),^24,25^ X-ray absorption spectroscopy (XAS),^26^ X-ray photoelectron spectroscopy (XPS),^27^ and more.^28−31^ Some of these methods were incorporated in HT electrochemical research.^32−34^*Operando* techniques for electrolyte analysis in electrochemical systems include inductively coupled plasma mass spectrometry (ICP-MS) and optical emission spectroscopy (ICP-OES),^35,36^ which can be easily coupled to electrochemical flow cell systems, making them an invaluable tool for electrocatalyst stability research.^36−38^

Depth of knowledge can conflict with the experimental throughput demonstrated by the platform, and the researcher must decide, depending on campaign objectives, where the optimum lies. It is essential to understand that the value of the experiment lies primarily in the quality of information gained from it and used to draw conclusions. The throughput should play a secondary role. Therefore, in HT campaigns, the throughput should be as high as possible while maintaining high quality of experiments and incorporating as much characterization as necessary.

## Quality

The quality aspect is broad and encompasses all essential components of the workflow, especially those directly contributing to data quality, such as material preparation and characterization. It is described by the control over the purity, morphology, and structure of synthesized materials, as well as measurement accuracy and precision. Regardless of HT workflows, it is crucial to the overall research quality to include quality control measures and metrics, specifically for platforms that can be employed in autonomous research where a researcher does not perform quality control themself. The reproducibility of experimental results plays a major part in this aspect.

## Accessibility of Techniques

An aspect describing how easy it is to gain access to techniques incorporated in a workflow. For example, synthesis by thermal precursor decomposition can be performed in most laboratories, while synchrotron measurements have very limited availability and are very geographically restricted. In another example, electrolyte analysis using ICP-MS is more accessible when performed *ex-situ*,^39^ compared to *in situ* measurements, which require coupling to flow cell systems.^40^ Accessibility is often limited by price. The capital as well as operational costs are often limiting factors in the transition from conventional to HT (and autonomous) experimentation. Fully automated characterization methods are often commercially available, although in a high price range, while the automation of conventional laboratory techniques requires highly specialized skills.

In principle, a fully automated workflow offers the possibility to be accessed by any researcher worldwide, which lowers the accessibility barrier for many techniques. Conventionally, technique accessibility is lowered by scientific collaborations, which are time-intensive and require the labor of skilled personnel. In fully autonomous laboratories, remote research could be conducted and evaluated with minimal human involvement.^41,42^

## Accessibility of Parameter Space

The parameter space defined for the scope of work may not be addressable with given techniques incorporated into the workflow. For example, the inherent limitations of the synthesis methods can restrict the obtainable morphology range and possible element combinations. In the case of analysis, detection limits may restrict the applicability of certain techniques. HT workflows addressing broad parameter spaces are highly desirable since they can be applied to a wide spectrum of experimental campaigns. A modular design of HT experimental setups facilitates this goal by enabling simplified platform modifications required by campaign-specific workflow adaptations.^42^ It is important to note that HT approaches can be applied to campaigns where parameters other than catalyst composition are varied. This can include, e.g., the effects of electrochemical parameters on reactions or synthesis method development.^43−45^

## Efficient Data Handling

Processing of the large amounts of data produced in HT workflows often constitutes a bottleneck of the entire process. Automating data acquisition and processing, as well as incorporating machine learning algorithms for data analysis and experimental guidance, can increase the experimental throughput and efficiency. Moreover, the (meta)data and code should be made available and easily accessible to allow for result reproduction, validation, and reuse. More information on community guidelines for data enabling is provided, e.g., by the FAIR principles.^10,46^

## Model Study on IrCo MMOs

HT workflows can be designed and evaluated using these aspects. Evaluation can facilitate the identification of potential areas for improvement. To demonstrate this possibility, we dissect a model study on IrCo MMOs in terms of the 6 aspects as we introduce the workflow and results.

The aim of the study was to rapidly assess the effect of Co addition on the activity and stability of Ir oxide catalysts for the acidic OER and identify the potential for future, more detailed studies. Concurrently, a novel strategy to enhance the reproducibility and quality of the drop-casting synthesis based on hydrogel substrate patterning was tested. With this motivation in mind, a previously reported HT workflow^47^ was adapted, which consisted of automated synthesis by drop-casting using a pipetting robot, HT quality control using a laser scanning microscope (LSM), and HT electrochemical activity and stability screening using a setup consisting of a scanning flow cell (SFC) coupled to ICP-MS. The workflow was expanded by HT compositional characterization using micro-X-ray fluorescence (μ-XRF), and two additional techniques with low throughput, scanning electron microscopy (SEM) and X-ray photoelectron spectroscopy (XPS), as it was found that they could provide essential information on the synthesis quality and the evolution of the catalytic surface due to electrochemical operation, respectively.

## Synthesis

The material library (ML) was prepared on a conductive fluorine-doped tin oxide (FTO) substrate by patterning it with a grid of drop-casted agarose spots, subsequent drop-casting of metal precursor inks on dried agarose spots and annealing in air. Agarose use in HT electrocatalyst synthesis was reported before, although not for patterning applications.^48,49^ All liquid handling operations, including ink transfer, mixing, and spotting, were performed by a pipetting robot in an automated manner, which contributes to the high throughput of this ML preparation method. Automation also results in increased spot reproducibility compared to drop-casting executed by humans, enhancing the quality aspect of the workflow. However, catalyst preparation by drop-casting and subsequent annealing generally produces materials of inferior quality compared to physical or chemical vapor deposition methods. On the other hand, the chosen synthesis method is highly accessible, compared to these more refined synthesis methods, due to the low price and low maintenance costs of the robot. A detailed synthesis procedure can be found in the Supporting Information. The choice of the synthesis method was motivated by the targeted catalyst morphology (thin films), which allows for direct screening with SFC-ICP-MS setups. However, other synthesis methods can be considered based on the campaign objectives.^50,51^

## Characterization

**Figure 1:**
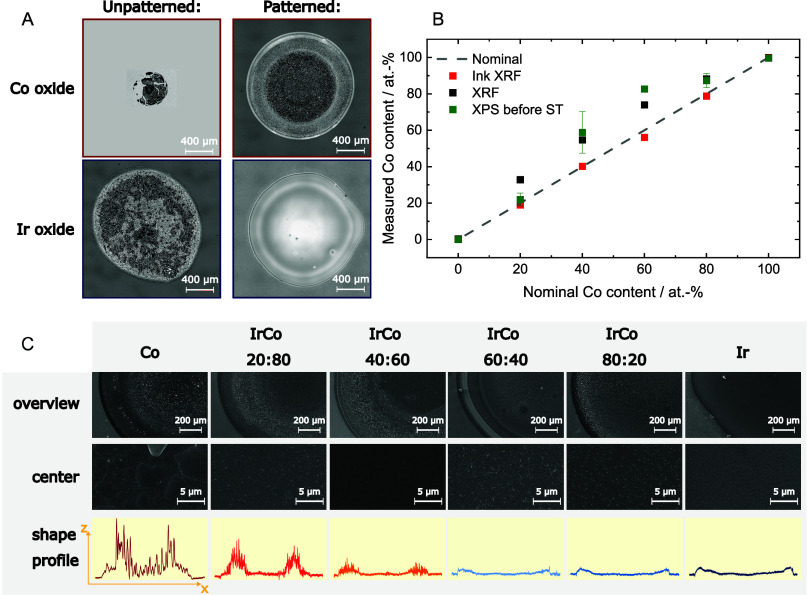
Catalyst characterization and quality assessment. LSM imaging of spots prepared without (unpatterned) and with (patterned) agarose (A). Compositional analysis of metal precursor inks and annealed spots with XRF and with XPS prior to stability testing (B). Error bars represent the standard deviation among replicates. SEM images of the spot composition spread recorded in secondary electron mode and spot shape profiles recorded with the LSM (C).

Two HT synthesis quality control steps were implemented in the workflow to assess the effect of agarose patterning on spot quality and synthesis reproducibility. LSM allowed for macroscale spot quality monitoring, including laser profiling, morphological homogeneity assessment, and extraction of geometrical spot areas using a custom Python script. LSM analysis showed improved spot morphological homogeneity and control over the shape and size of the spots, independent of the metal precursor used in the ink (Figure 1A, 1C, and Supporting Information). A second quality control step, involving compositional analysis by μXRF, was used to validate the synthesis accuracy by comparing the composition of precursor inks used for ML fabrication with the composition of annealed spots (Figure 1B). Interestingly, the nominal ink compositions were not reflected in the final spot composition after thermal annealing, which highlights the importance of quality control in workflow design to avoid false property assignment to nominal compositions. Two additional analyses with lower throughput were performed to validate the microscale morphology of patterned spots (SEM) and to assess the catalyst surface compositions before and after metal dissolution screening (XPS). It should be noted that commercial technical solutions that increase the throughput of SEM and XPS exist, although with limited availability due to the high price.^52^ SEM images were performed in secondary electron (SE) mode to show the surface microscale morphology (Figure 1C) and backscattered electron (BE) mode to investigate metal mixing (Figure S1) in the microscale. Spot images over a (nominally) 20 at.-% composition spread show lower homogeneity in Co-rich spots, likely caused by the design of the annealing process (more rapid decomposition of the Co precursor compared to Ir precursor). Images recorded in BE mode show uniform metal distribution in the measured length scales. As OER occurs on the catalyst surface and in its vicinity, surface-sensitive XPS analysis was performed to address the spot compositions and their evolution during metal dissolution testing.^53^ The compositions measured prior to the dissolution study did not precisely match the bulk compositions, suggesting compositional inhomogeneities throughout the spot thickness (Figure 1B). For clarity, the samples are named according to the nominal relative metal ratio in the samples in the following discussion, where, e.g., IrCo 80:20 corresponds to a sample with nominally 80 at.-% Ir relative to Co.

Synthesis by drop-casting shows morphological and compositional inhomogeneities, which negatively affect the quality aspect of the workflow although an improvement in spot quality was achieved with agarose patterning. The accessibility of the parameter space is affected as well since the target compositions were not achieved. These findings suggest caution with thermal precursor decomposition processes. This synthesis may not be suitable for studies aiming at precise fundamental investigations of highly uniform materials, although it is useful for rapid preliminary screening, particularly with quality control measures. Quality control may lower the throughput of the workflow. However, its application is mandatory to ensure reliable data output. As for incorporating low-throughput characterization methods, one must consider the gain in depth of knowledge they offer with regard to the experimental campaign objectives. In the case of the model study, expanding the workflow by relatively accessible SEM and XPS methods provided valuable insights into the synthesis quality, justifying the prolongation of the overall study. The data processing in this part of the study was not automated, which will be addressed in further workflow development.

## Electrochemical Screening

An ideal catalyst is both active and stable under operating conditions. Therefore, both parameters should be screened to properly assess the potential of lowering iridium loading by introducing cobalt into the acidic OER catalyst. A system composed of an SFC coupled to ICP-MS was chosen for the model study due to the invaluable depth of knowledge it offers for electrochemical stability research by simultaneous time-resolved electrolyte composition analysis and electrochemical data collection. Due to a high degree of automation, the system performs serial measurements in an HT manner. A detailed description of the setup can be found elsewhere.^47^ The setup allows for tuning the measurement throughput and depth of knowledge according to the campaign objectives. The technique has limited accessibility due to its price, complexity, and lack of off-the-shelf availability. Such setups are typically custom-built, which allows a modular, flexible design. Regarding the accessibility of parameter space, SFC-ICP-MS setups score high due to the broad range of elements detectable with ICP-MS, the possibility of quantifying the dissolution products with isotopic resolution, and extremely low detection limits.^35^

## OER Activity

**Figure 2:**
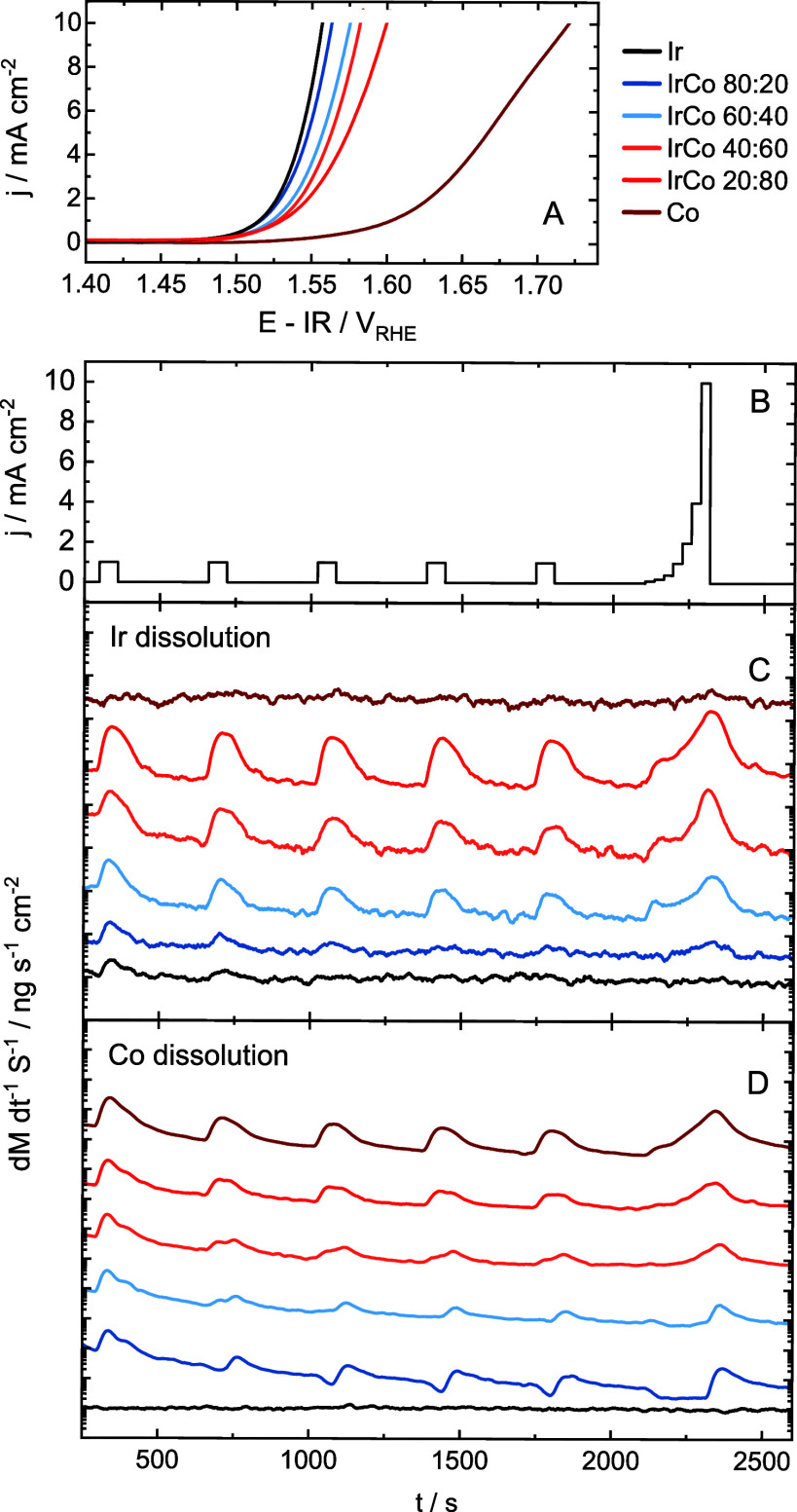
HT electrochemical measurements. LSV curves showing the OER activity trend over the composition spread (A). Applied electrochemical protocol (B) and the resulting metal dissolution profiles for Ir (C) and Co (D). Dissolution profiles are shown in linear scale in Figure S4.

Rapid screening of the catalyst activity was performed using linear sweep voltammetry (LSV) for all compositions. The measured activity trend (Figure 2A) shows a significantly lower activity of pure cobalt oxide compared to other compositions, while the activity increases with increasing Ir loading. The activity data was additionally normalized by capacitance measured with cyclic voltammetry to assess the effect of current normalization on the observed activity trends. These results and a discussion about activity reporting can be found in the Supporting Information (Figures S2, S3, and section 5 of the Supporting Information). There is a clear beneficial influence of Ir on Co. However, what remains to be determined is whether Co is an active component or the activity of Ir oxide is simply decreased due to lowering the amount of Ir active sites when diluting with Co. The activity of pure Ir oxide was not exceeded by MMO compositions, although a beneficial electronic influence of Co on Ir cannot be ruled out. More insights into the electronic effects of Co addition should be further investigated.

## Metal Dissolution

Having identified the activity trend and the relatively high OER activities of MMOs, the next step was to assess the stability in terms of metal dissolution. To simulate the fluctuating power of water electrolyzers powered by renewable energy sources, an electrochemical protocol consisting of open circuit potential (OCP), and five consecutive chronopotentiometric (CP) holds at 1 mA cm^–2^ separated by idle breaks at OCP was applied. This was followed by a CP staircase hold consisting of a sequence of holds at 0.1, 0.2, 0.4, 1, 2, 4, and 10 mA cm^–2^, and OCP (Figure 2B), allowing for Tafel analysis at a semisteady state at each applied current density.

There are several ways in which the metal dissolution characteristics can help understand the stability of the investigated system. Essential information can be gained from analyzing dissolution profiles and their integrals. The profiles for Ir and Co across the composition spread are shown in Figure 2C and 2D, respectively.

**Figure 3:**
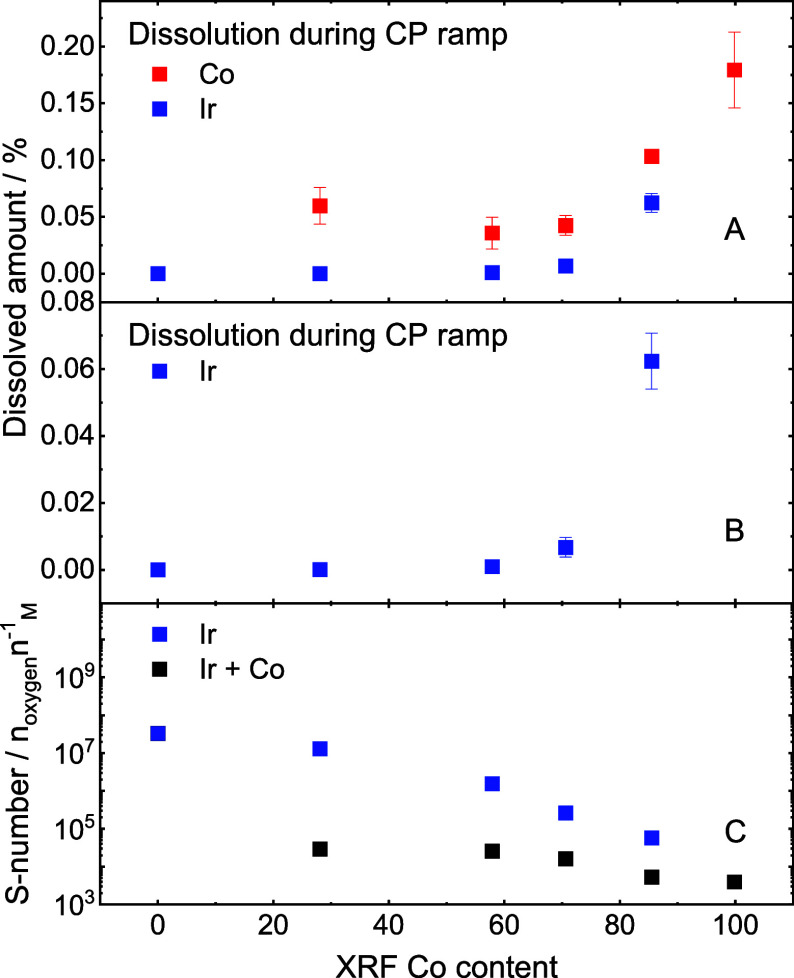
Results of metal dissolution experiments. Dissolved amount of metal normalized by total metal loading per spot for both metals (A), and magnified trend for Ir only (B). Stability numbers considering Ir dissolution and the combined dissolution of Ir and Co during the CP ramp (C).

What follows is an example of a rather detailed analysis with much human involvement. However, the data processing can be automated. In the case of this study, the automation was partial, as only some steps of data processing were performed with custom LabVIEW programs. Regarding the quality aspect, all SFC-ICP-MS experiments were replicated, and the variability is shown through error bars in Figure 3. The results were reproducible within the testing platform, and increased variability was observed as a function of composition, where Co-rich samples had more diverse performance among replicates.

The scope of information that can be extracted from *operando* dissolution experiments is rich and ranges from fundamental insights, such as dissolution mechanisms (an analysis of the mechanisms of dissolution can be found in the Supporting Information), to simple stability assessments. Quantifying the number of dissolved species allows for comparing metal oxide stabilities. For this purpose, the amounts of dissolved metals normalized by metal loading per spot are shown in Figure 3A, and a magnification of the Ir dissolution trend is shown separately in Figure 3B due to very low Ir dissolution.

As expected, Ir is far more stable than Co, with stability decreasing for more Co-rich compositions. Intensified dissolution of iridium in the presence of non-noble metals in iridium MMOs is a known phenomenon and can be due to changes in lattice spacing and oxidation states of metals introduced by mixing different metals.^15,17,54,55^ Moreover, the less stable element may dissolve around the more stable one, leading to its loss.

On the other hand, Co is stabilized by Ir, where Co dissolution is decreasing with the growing Ir content. The increased Co dissolution in the IrCo 80:20 sample is caused by the larger cathodic dissolution peak, as the anodic dissolution peak is absent. Ir dissolution increases strongly when exceeding 60 at.-% Co. However, it remains within 10^–4^ mass% at lower Co loadings. The nonlinear trend of Ir dissolution can be explained by higher Ir-content on the surface of the catalysts, as suggested by XPS measurements before and after the protocol in Figure S6.

Quantifying the dissolution of OER active metals offers the possibility to assess the overall stability of the catalysts with the stability number (S-number), which is a dimensionless metric used to assess the amount of evolved oxygen per the amount of dissolved active metal detected with the ICP-MS, assuming 100% faradaic efficiency toward oxygen evolution (Figure 3C). This metric is independent of the surface area and loading, and a high S-number indicates a stable catalyst.^56^ Here, both Ir dissolution and the combined dissolution of both metals were considered. The total integrated charge over the CP ramp was normalized by the amount of Ir, and the sum of Ir and Co dissolved. The S-numbers for both Ir and IrCo MMOs decrease with increasing Co loading. The high Co dissolution is the main contributor to the low MMO S-numbers. ASTs of Ir-rich compositions would be an interesting follow-up to the presented work to investigate whether the catalyst composition eventually stabilizes after leaching out excess Co since Co dissolution decreases with time (Figure 2D). It should be noted that the catalyst activity and stability determined in simplified model systems such as SFC or rotating disc electrode are often not directly transferrable to the real device level. HT studies approaching real operating conditions remain a challenge, although solutions such as scanning gas diffusion electrode setups are emerging.^57^ Therefore, although useful as a performance metric on any level, the S-number should be validated in real operating conditions.^58^ An additional analysis of changes to activity due to dissolution and Tafel slope trend over composition can be found in Supporting Information.

Finally, we shortly summarize the evaluation of the applied workflow in terms of the previously outlined aspects for the design of HT workflows in the service of electrocatalytic research. The throughput of the synthesis as well as characterization, including LSM, XRF, and SFC-ICP-MS, was high due to parallel synthesis, high level of automation of the mentioned techniques, and considering the richness of information gained from a single set of SFC-ICP-MS measurements. Additional characterization techniques, including SEM and XPS, were of lower throughput. However, the process bottleneck was data processing, resulting in a low score in the efficient data handling aspect. Regarding the depth-of-knowledge aspect, the information produced in the workflow allowed for meeting the study’s objectives, motivating future investigations into IrCo MMOs. The quality of the synthesis method was improved by the agarose patterning method, yielding samples of reproducible properties, although the predefined compositional space of interest was changed due to inaccuracy in synthesis. The accessibility of the workflow is moderate due to the incorporation of several costly techniques, most of which, however, are standard in materials research laboratories.

Future improvements to the presented HT workflow will focus on gaining more control over the synthesis process and attempts to introduce automation to XRF characterization. Moreover, a shift to a data management system resembling that reported by Röttcher et al., which allows for HT data storage, processing, and communication in accordance with the FAIR principles, is planned.^59^ For investigations of more complex systems, introducing machine learning for experimental guidance will obtain greater attention.^7^

## Data Availability

Experimental data used in the model study is available at the following link: https://github.com/jmprzybysz/Research-Data/tree/main/Key-Aspects

## Supporting Information Available

Experimental methods, supplementary figures, additional remarks and analyses (incl. metal dissolution profiles and Tafel analysis), discussion on activity reporting (PDF)
