# Supplementary material for: Key Aspects in Designing High-Throughput Workflows in Electrocatalysis Research: A Case Study on IrCo Mixed-Metal Oxides
Source: ACS Mater Lett. 2024 Oct 15;6(11):5103–11. doi: 10.1021/acsmaterialslett.4c01372 (PMC11539082; doi:10.1021/acsmaterialslett.4c01372)
Supplement: Supplementary file 1 — tz4c01372_si_001.pdf [file tz4c01372_si_001.pdf]

Supplementary Information

Key Aspects in Designing High-Throughput Workflows in Electrocatalysis Research: A Case Study on IrCo Mixed-Metal Oxides

Joanna M. Przybysz,[1*] Ken J. Jenewein,[1] Mária Minichová,[1] Tomáš Hrbek,[2] Thomas Böhm,[1] Tatiana Priamushko,[1] Serhiy Cherevko[1*]

[1] Helmholtz-Institute Erlangen-Nürnberg for Renewable Energy (IET-2), Forschungszentrum Jülich, Cauerstrasse 1, 91058 Erlangen, Germany

[2] Charles University, Faculty of Mathematics and Physics, Department of Surface and Plasma Science, V Holešovičkách 2, 180 00 Prague 8, Czech Republic

*Corresponding authors: j.przybysz@fz-juelich.de , s.cherevko@fz-juelich.de

1. Experimental methods

FTO cleaning and hydrophobization

The FTOs were sonicated for 5 minutes in the following solutions: Hellmanex solution 2 vol.-% (Hellma), ultrapure water (miliQ), and isopropanol (Sigma Aldrich, ACS reagent, ≥99.8%). After drying in nitrogen gas, the FTOs were placed in a 6 vol.-% solution of dichlorodimethylsilane (Sigma Aldrich, >99.5%) in hexane (Sigma Aldrich, ReagentPlus, ≥99%) for 5 minutes, rinsed with hexane and dried with nitrogen gas. Silanization was performed to hydrophobize the electrode surface for improved agarose droplet shape retention. In unpatterned synthesis, this step is also used for local containment of ink droplets.[1]

Metal precursor ink preparation

Metal precursor inks were prepared with 1 M stock solutions of metal precursors ($Co(NO_3)_2 \cdot 6H_2O$ (Sigma Aldrich, 98%), $H_2Cl_6Ir \cdot xH_2O$ (Sigma Aldrich, 99.9%)), a stock solution of 70 vol.-% glycerol (Sigma Aldrich ≥99.5%) in ultrapure water and ultrapure water. To prepare a 15 mM ink, 15 µl of respective 1 M precursor stock solution was mixed on a vortex with 428.7 µl of the glycerol stock solution and 556.3 µl of ultrapure water in a 1.5 ml tube (Eppendorf).

Preparation of the agarose solution and automated agarose drop-casting

A stock solution of 0.5 wt.-% agarose was prepared by dissolving 15 mg of agarose powder (Sigma Aldrich, medium EEO, ≤10% moisture content) in 2.985 g of ultrapure water in 70 °C on a hot plate with magnetic stirring until complete agarose dissolution was observed. After cooling down, solutions of 0.05 wt.-% agarose were prepared by diluting the stock solution. These solutions were then transferred to the solution handling robot (epMotion 5073, Eppendorf) and were drop-casted onto cleaned and silanized FTOs held in custom-designed holders. 1 µl of 0.05 wt-% agarose solution was transferred per spot. Each FTO was patterned with a 9x9 grid.

Material library (ML) preparation

The sequential liquid transfer, mixing of inks for the composition spread, and spotting operations were handled by the pipetting robot. To prepare inks with a composition spread, 4 µl, 8 µl, 12 µl, 16 µl and 20 µl of each precursor ink were transferred into a multi-well plate. The total ink volume in each well was set to 20 µl, and the inks were prepared with a 20 at.-% spread. 3 µl of ink with a given composition were transferred per agarose spot. Typically, 9 spots of each composition were synthesized. The drop-casted MLs were annealed in a tube furnace (GHA 12/300, Gero Carbolite) for 2 hours at 500 °C, in air, with a heating rate of 1 K min^{-1}.

Quality control – Laser Scanning Microscope (LSM)

The homogeneity, shape profiles, and size of annealed spots were investigated using an LSM (VK-X250, Keyence). Images of ML overviews and individual spots were recorded with 5x magnification in assembly mode. For shape profile measurements, 20x magnification was used for improved accuracy.

Scanning electron microscopy (SEM)

SEM imaging of the samples was performed using a Tescan Vega 3 scanning electron microscope, operated at 20 kV accelerating voltage. Images were recorded with the secondary electron detector and the backscattered electron detector. One spot was measured per composition.

X-ray fluorescence spectroscopy (XRF)

Compositional analysis was performed with a Bruker M4 Tornado micro-XRF spectrometer with a rhodium X-ray source operated at 50 kV and 600 μA current and a 20 μm beam size. The device was operated at 20 mbar pressure, and Co K-α (6.9 keV) and Ir L-α (9.2 keV) lines were used for element quantification. Spots were analyzed with 20-point area scans with a 25 s measurement time per spot. The composition of concentrated 1 M metal precursor inks spotted on FTO was measured with three randomly chosen point scans over each droplet area. Two spots were measured for each composition.

X-ray photoelectron spectroscopy (XPS)

The XPS measurements were conducted using an EnviroESCA device (SPECS) with a monochromatized Al Kα source (1486.71 eV). The signal detection was performed using a Phoibos 150 NAP 1D-DLD (SPECS) hemispherical analyzer in the Fixed Analyzer Transmission (FAT) regime. The EnviroESCA device allows for precise choice and measurement of a 300 μm^2 area, which allows selecting spots used for electrochemical analysis. The number of measurement sets was set for each element individually to minimize the signal-to-noise ratio. Four spots were measured for each composition, including two measurements of as prepared spots and two measurements of spots after stability testing.

SFC measurements

All electrochemical measurements were performed using an SFC setup coupled to ICP-MS for on-line stability measurements. In an SFC, the scanning cell is mounted above an xyz-translational stage housing a working electrode. It is connected via tubing with an Ag|AgCl|3 M KCl reference electrode on the cell outlet and a glassy carbon counter electrode (SIGRADUR G, HTW) on the cell inlet. A separate channel in the cell delivers argon gas around the measured area of the working electrode. The electrolyte flow was controlled using a peristaltic pump (Reglo ICC, Ismatec). The electrolyte was purged with argon gas in a purging reservoir prior to entering the SFC. All elements of the system were connected by Tygon tubing.

All hardware was controlled using software developed in-house, which enables measurement automation. Electrochemical measurements were performed using a potentiostat (Ref600, Gamry). After initial calibration of the position of the first spot (located in the upper left corner of the material library) by alignment with the SFC opening, the SFC is capable of accurate navigation over the whole ML using spot coordinates extracted from ML overview images recorded using the LSM with a Python script and loaded into the SFC software. A detailed description of the automated SFC(-ICP-MS) operation can be found in the following reference.[1]

All electrochemical potentials reported in this work were recalculated against the reversible hydrogen electrode (RHE). The potential of the Ag|AgCl|3 M KCl reference electrode against the RHE was measured prior to every set of experiments. Full iR compensation was applied to the presented data. The iR drop was measured using electrochemical impedance spectroscopy at 300 mV with an AC sinusoidal signal with a 20 mV amplitude in the frequency range of 10 kHz to 100 Hz. Three locations on the FTO plate were measured using the SFC in a 0.05 M H_2SO_4 electrolyte. The average value was used for the iR drop correction of electrochemical measurements.

For the LSV activity screening, three LSVs up to 10 mA cm^{-2} were recorded for the composition spread in 0.05 M H_2SO_4. The third LSV was used for analysis. Three spots were measured for each composition.

The material capacitance was obtained by measuring CVs at 50 mV s^{-1}, 100 mV s^{-1}, and 200 mV s^{-1} scan rates in a 0.4 – 1.4 V_{RHE} potential window in 0.05 M H_2SO_4, extracting capacitive current at 0.5 V_{RHE} and 1.2 V_{RHE}, plotting it over the scan rate and extracting the slope of the obtained linear function $i_c = C\nu$, where i_c is capacitive current, C is capacitance and ν is the scan rate.[2-4] Exemplary CVs of pure Co oxide and pure Ir oxide spots are shown in Figure S2. The used capacitive current was half of the net difference between the anodic and cathodic sweep at a given potential.

SFC-ICP-MS measurements

The combined activity and stability measurements were performed using the SFC coupled to an ICP-MS (Nexion 350X, PerkinElmer) by Tygon tubing, where all outflowing electrolyte was introduced into the ICP-MS. The ICP-MS was optimized daily, prior to the measurements, using a NexION Setup Solution (Perkin Elmer), and the dissolution signals of ^{193}Ir and ^{59}Co were quantified using a four-point calibration curve (0, 0.5, 1, 5 µg L^{-1}). Calibration solutions were prepared daily. ^{187}Re and ^{74}Ge were used as internal standards at a 10 µg L^{-1} concentration (Certipur ICP-MS Standard, Merck).

The measurements were conducted in 0.05 M H_2SO_4 electrolyte, with a galvanostatic protocol composed of a hold at open circuit potential (OCP) for 300s, 5x CP holds @ 1 mA cm^{-2}_{geom}, each one for 60s, followed by 60s at open circuit potential (OCP), a CP ramp consisting of a sequence of 30s CP holds at 0.1, 0.2, 0.4, 1, 2, 4, and 10 mA cm^{-2}, and OCP. The chosen low current densities allow for stability screening within reasonable measurement time and prevent overloads that might occur due to bubble formation during measurements. Two spots were measured for each composition.

2. Supplementary figures

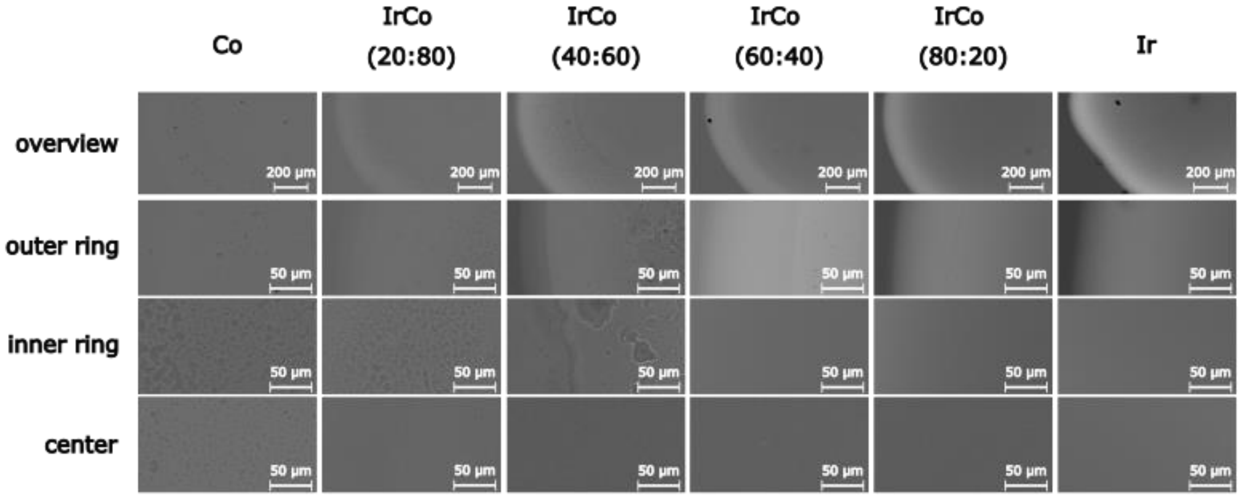

Figure S1: SEM images of the ML recorded in backscatter electron mode. Three regions were identified on the spots based on morphological inhomogeneities observed on SEM images recorded in the secondary electron mode: spot center, inner ring, and outer ring.

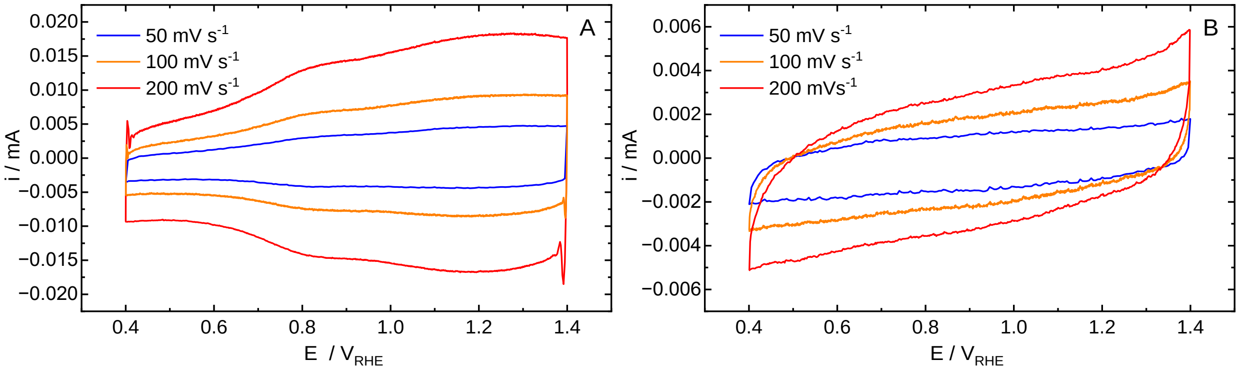

Figure S2: Exemplary cyclic voltammograms of Ir oxide (A) and Co oxide (B) used for the determination of capacitance, recorded in 0.05 M H_2SO_4 electrolyte at 50 mV s^{-1}, 100 mV s^{-1} and 200 mV s^{-1} scan rates.

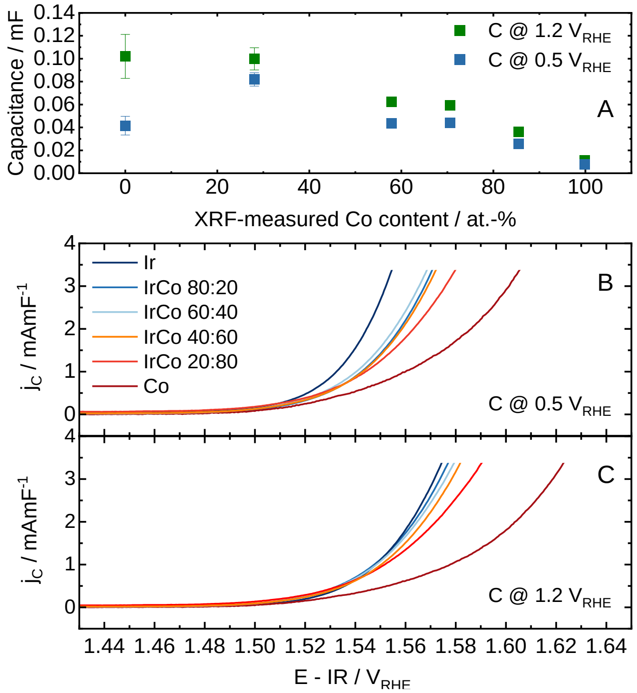

Figure S3: Capacitance trend over the composition spread, where the capacitive current was extracted at 0.5 V_{RHE} and 1.2 V_{RHE}(A). OER activity over the composition spread with current normalized by capacitance, where the capacitive current was extracted at 0.5 V_{RHE} (B) and 1.2 V_{RHE} (C).

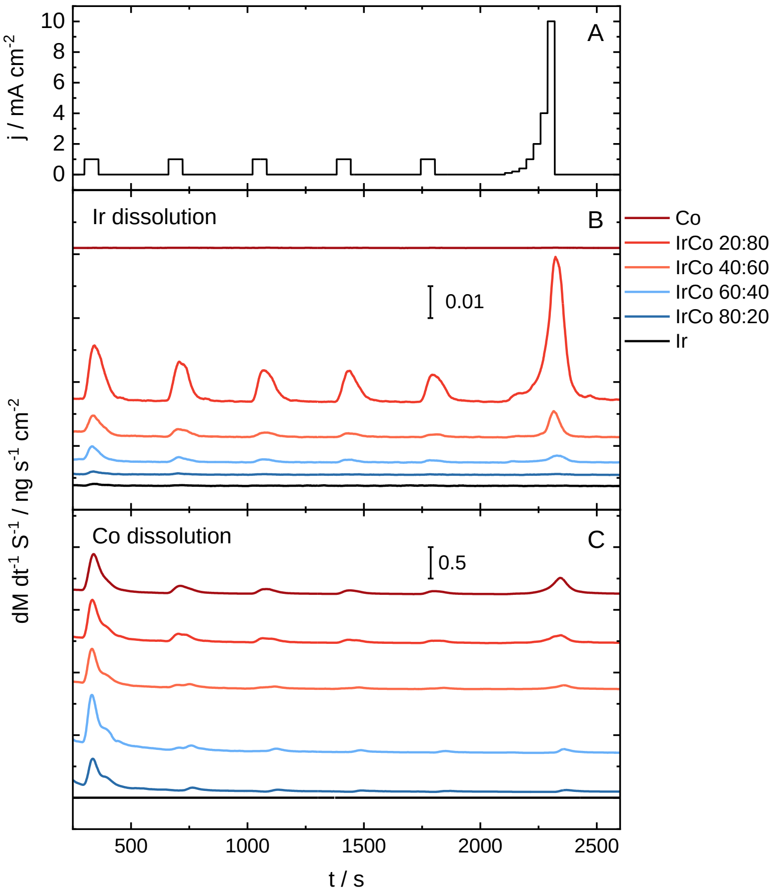

Figure S4: HT electrochemical measurements. Applied electrochemical protocol (A) and the resulting metal dissolution profiles for Ir (B) and Co (C).

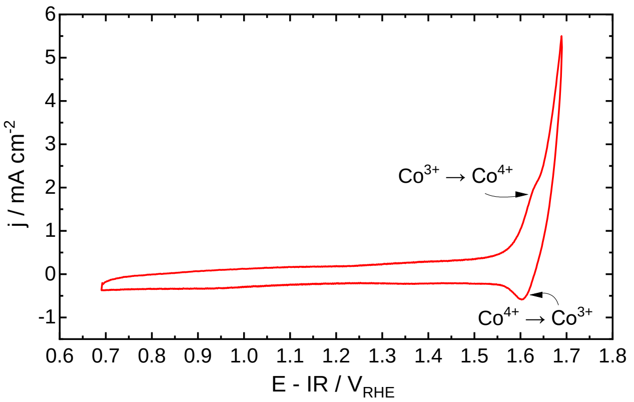

Figure S5: Cyclic voltammogram of pure Co oxide spot recorded at 200 mA cm^{-2} scan rate, in 0.05 M H_2SO_4. iR drop correction was applied.

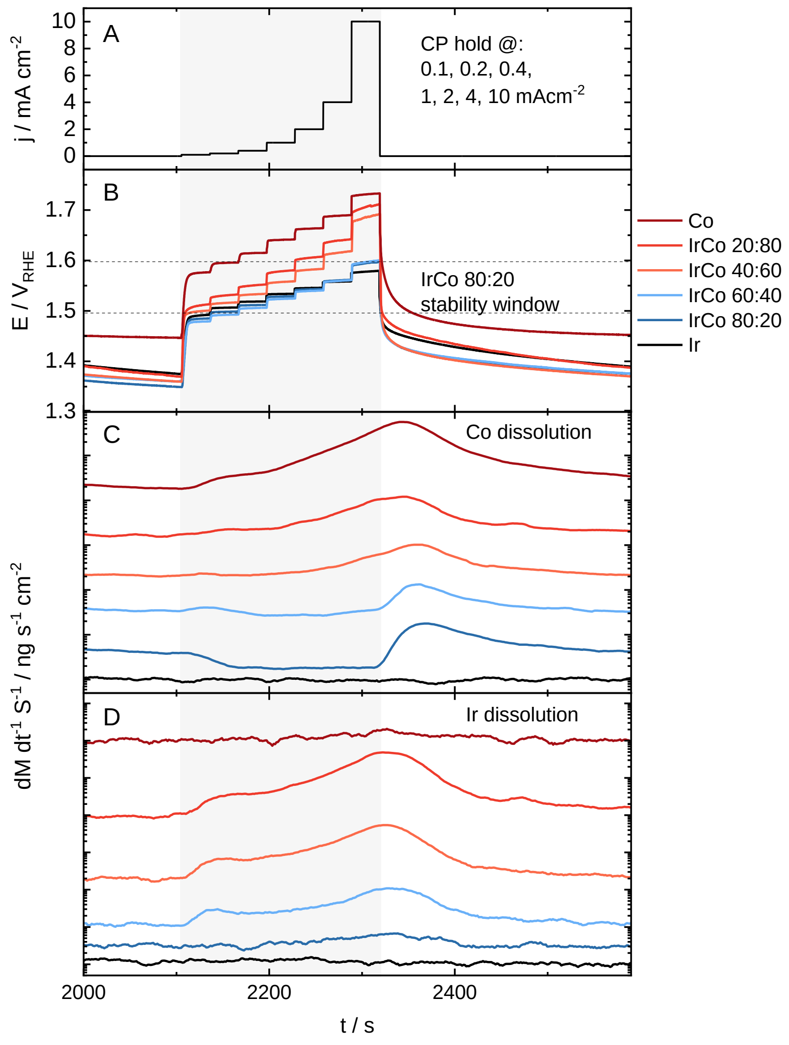

Figure S6: Applied current (A), potential response (B), and dissolution profiles of Co (C) and Ir (D) across the composition spread.

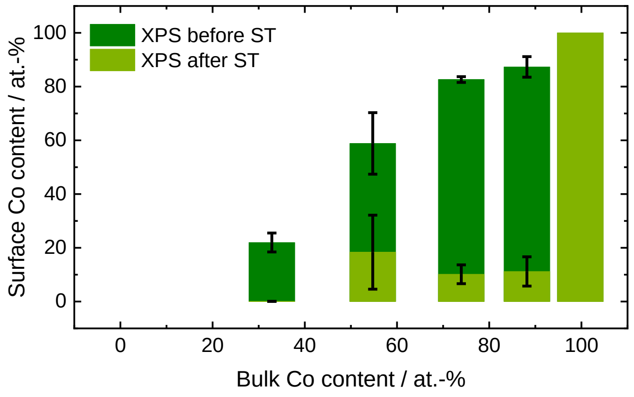

Figure S7: XPS-measured Co relative content vs. Ir over the composition spread before and after stability tests (ST).

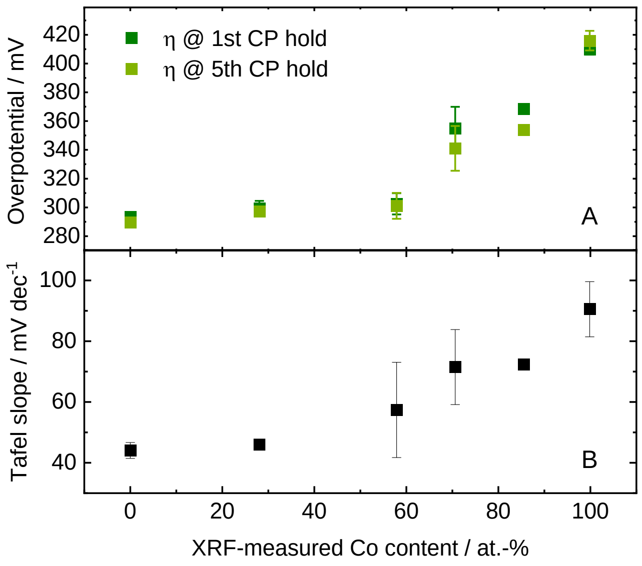

Figure S8: Overpotentials extracted at the first and fifth CP holds at 1 mA cm^{-2} (A). Tafel slope values extracted from Figure S9 (B). Error bars refer to the standard deviation across sample replicates. All values plotted against the XRF-measured Co content relative to Ir.

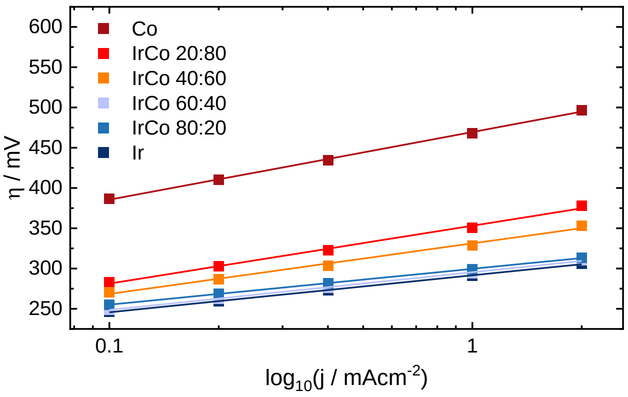

Figure S9: Tafel analysis plot for the composition spread.

3. Additional remarks on patterned drop-casting synthesis

The patterned spots showed very good adherence to the substrate, as opposed to spots prepared without agarose. Laser profiling revealed a coffee-ring deposition pattern occurring in all spots, which is typical to the synthesis by drop-casting and causes radially changing morphological features due to thickness variation present in synthesized spots, which can be observed in LSM and SEM images (Figure 1A and C). The spot thickness variation introduced by the coffee-ring deposition pattern can affect the spot OER activity due to conductivity changes. The effect of Co oxide film thickness on OER activity has been previously reported by Nie et al..[5]

4. Changes to OER activity over CP holds and Tafel analysis

To address the question how OER activity is affected by the changes to the catalyst structure caused by metal dissolution, we compare the overpotentials extracted at the first and fifth CP holds at 1 mA cm^{-2} for the composition spread (Figure S8A). The overpotentials are comparable at both CP holds for most compositions, indicating no significant loss of activity. Longer stability testing or applying higher current densities is required to observe changes in activity. Generally, the overpotential increases with Co loading, resembling an exponential trend in activity loss, which is also reflected in the Tafel slope analysis (Figure S8B, Figure S9) based on the CP ramp at the end of the protocol. The increase in overpotentials and Tafel slope with higher Co content is expected due to the lower intrinsic OER activity of Co compared to Ir. The values of the Tafel slope for Co oxide and Ir oxide are comparable to literature values for thin film catalysts (approx. 80 mV dec^{-1} as reported by Mondschein et al. and 40-50 mV dec^{-1} as reported by Cherevko et al., respectively).[6, 7] The similarity in trends for activity and Tafel slope across the composition spread suggests that a large contribution to the activity loss with Co addition is of kinetic origin. The activity trend observed in the Tafel slope analysis is likely caused by the major Co dissolution in MMOs during the stability testing (Figure S7), where the Co relative content for most MMO compositions reaches around 10-20 Co at.-%. The Co content in the most Ir-rich MMO is reduced to about 0.1 at.-%. These results suggest that the effect of Co addition is purely diluting Ir and lowering its activity in the investigated compositions.

5. On activity reporting and normalization by capacitance

Due to the very different nature of Ir and Co, and the resulting differences in thin film morphology, it must be considered whether normalization by geometrical area is a reliable way to report electrochemical activity. The geometrical area does not consider the actual electroactive surface area of the catalysts and neglects the morphology and particle size of catalyst surfaces.[8] Therefore, it does not accurately reflect the intrinsic activity of the material under investigation.[3, 4, 9] Therefore, to compare the activity trend across the composition spread, we additionally choose to normalize the current in LSV measurements by capacitance, which serves as a proxy metric relating to the catalyst's electrochemically active surface area. In general, proxy metrics are experimentally assessed properties, indirectly representing properties that are difficult to measure. In this case, capacitance represents the electroactive surface area of the metal oxide materials, for

which a standardized, accurate, and rapid assessment method does not exist to date. Commonly, the measured capacitance is converted to surface area using specific capacitance. However, these values are usually assumed to be a certain value extracted from literature, which leads to errors. Thus, we avoid this conversion step to reduce the error in estimating the electroactive surface area.

The change in measured capacitance over the composition spread is presented in Figure S3A for two potentials of capacitive current extraction. The current was extracted at 0.5 V_{RHE} and 1.2 V_{RHE}, where at the latter potential, the contribution of pseudocapacitive current for Ir oxide is larger. OER on hydrous Ir oxide was previously reported to be a three-dimensional structure, where active sites within the material bulk, specifically the hydrous Ir oxide layer, contribute to the activity.[10, 11] Therefore, considering the pseudocapacitive current for activity normalization by capacitance may be more representative of Ir MMO OER activity. The capacitance increases with Ir content, with close values for pure Ir oxide and the IrCo (80:20) sample, when capacitance is extracted at 1.2 V_{RHE}. The trend breaks down at pure Ir oxide when capacitance is extracted at 0.5 V_{RHE}. The resulting trend in activity for i_C extracted at 0.5 V_{RHE} (Figure S3B) shows overlapping CVs for the MMO compositions and a significantly higher activity of Ir oxide over other compositions. The low value for pure Ir oxide can be due to a particularly smooth surface, as observed in SEM images and surface profiles (Figure 1C), hence lower electroactive surface area. The trend in capacitance over the composition spread for capacitance extracted at 1.2 V_{RHE} follows that of the activity trend where the geometrical area was used for normalization (Figure S3C). The high activity maintained for MMOs with high Co loadings is probably not due to an increase in the surface area since the capacitance increases with Ir content (when i_C is extracted at 1.2 V_{RHE}) and changes almost linearly over the composition spread (Figure S3A). However, this trend could result from the large contribution of pseudocapacitive current to the measured capacitance.[9, 12]

The activity trend is unclear. However, valuable conclusions are valid in the case of all applied normalization methods. The performance of Ir oxide catalyst is not enhanced with the addition of Co, and with a tradeoff between activity and material cost, it is still promising to consider diluting Ir with Co in MMOs, as the MMO compositions show relatively high OER activity.

6. Analysis of metal dissolution profiles

A single anodic dissolution peak was identified for Ir, caused by oxidation of Ir to higher oxidation states and OER, when OER onset potentials are reached around 1.5 V_{RHE} (Figure S2 and Figure 2).[13] In the case of Co, two distinct dissolution peaks are identified, one anodic, with an onset aligned with the onset of CP holds, and one cathodic, after return to OCP. Both are fully merged into one peak for pure Co oxide spots caused by high dissolution. The anodic peak is due to Co oxidation to higher oxidation states (see Figure S5) and OER. The cathodic peak is caused by reverse, reductive reactions. Anodic Co dissolution peaks decrease in intensity, while cathodic peaks become more enhanced with increasing Ir content and the number of applied CP holds. The first anodic CP hold dissolution peak is larger than those consecutive, likely due to oxidation of Co(II) species present on the catalyst surface.[14] Most of these surface species get oxidized during

the first CP hold, which translates to a decrease in the anodic peak intensity with consecutive CP holds.

In the dissolution profile of the IrCo 80:20 sample, the anodic peak seems to have completely vanished, which suggests the existence of a potential stability window. An expanded view of the dissolution behavior during the CP ramp and corresponding potential profiles across the ML are shown in Figure S6. The stability window is identified in the potential window of approximately 1.5 V_{RHE} to 1.6 V_{RHE}. A stability window possibly exists for Co-rich compositions as well, although it is likely masked by the dissolution peak caused by transient processes at the onset of the CP ramp, which increases in intensity with the amount of Co loading in a spot. It is, therefore, not possible to state the potential range of such a window for these compositions with the applied protocol, and additional investigations should follow. Baseline (chemical) dissolution and total dissolution per CP hold decreases with time for both metals and all compositions. The dissolution peak, which occurs consequently to the initial contact of the electrode with the electrolyte (contact peak), is not shown for clarity.

References

(1) Jenewein, K. J.; Akkoc, G. D.; Kormányos, A.; Cherevko, S. Automated high-throughput activity and stability screening of electrocatalysts. *Chem Catalysis* **2022**, *2*, 2778–2794. DOI: 10.1016/j.checat.2022.09.019.
(2) McCrory, C. C. L.; Jung, S.; Peters, J. C.; Jaramillo, T. F. Benchmarking Heterogeneous Electrocatalysts for the Oxygen Evolution Reaction. *Journal of the American Chemical Society* **2013**, *135*, 16977-16987. DOI: 10.1021/ja407115p.
(3) Trasatti, S.; Petrii, O. A. Real surface area measurements in electrochemistry. *Journal of Electroanalytical Chemistry* **1992**, *327*, 353-376. DOI: 10.1016/0022-0728(92)80162-W.
(4) Wei, C.; Sun, S.; Mandler, D.; Wang, X.; Qiao, S. Z.; Xu, Z. J. Approaches for measuring the surface areas of metal oxide electrocatalysts for determining their intrinsic electrocatalytic activity. *Chemical Society Reviews* **2019**, *48*, 2518-2534, 10.1039/C8CS00848E. DOI: 10.1039/C8CS00848E.
(5) Nie, Z.; Zhang, B.; Zhang, J.; Hu, K.; Ma, G.; Yang, N. The Role of Cobalt-Based Cocatalysts on BiVO 4 for Photoelectrochemical Water Oxidation. *ChemCatChem* **2024**. DOI: 10.1002/cctc.202301683.
(6) Mondschein, J. S.; Callejas, J. F.; Read, C. G.; Chen, J. Y. C.; Holder, C. F.; Badding, C. K.; Schaak, R. E. Crystalline Cobalt Oxide Films for Sustained Electrocatalytic Oxygen Evolution under Strongly Acidic Conditions. *Chemistry of Materials* **2017**, *29*, 950–957. DOI: 10.1021/acs.chemmater.6b02879.
(7) Cherevko, S.; Geiger, S.; Kasian, O.; Mingers, A.; Mayrhofer, K. J. J. Oxygen evolution activity and stability of iridium in acidic media. Part 2. – Electrochemically grown hydrous iridium oxide. *Journal of Electroanalytical Chemistry* **2016**, *774*, 102–110. DOI: 10.1016/j.jelechem.2016.05.015.
(8) Morales, D. M.; Risch, M. Seven steps to reliable cyclic voltammetry measurements for the determination of double layer capacitance. *Journal of Physics: Energy* **2021**, *3*, 034013. DOI: 10.1088/2515-7655/abee33.
(9) Wei, C.; Rao, R. R.; Peng, J.; Huang, B.; Stephens, I. E. L.; Risch, M.; Xu, Z. J.; Shao-Horn, Y. Recommended Practices and Benchmark Activity for Hydrogen and Oxygen Electrocatalysis in Water Splitting and Fuel Cells. *Adv Mater* **2019**, *31*, e1806296. DOI: 10.1002/adma.201806296.
(10) Burke, L. D.; O'Sullivan, E. J. M. Oxygen gas evolution on hydrous oxides — An example of three-dimensional electrocatalysis? *Journal of Electroanalytical Chemistry and Interfacial Electrochemistry* **1981**, *117*, 155–160. DOI: 10.1016/s0022-0728(81)80459-7.
(11) Cherevko, S.; Reier, T.; Zeradjanin, A. R.; Pawolek, Z.; Strasser, P.; Mayrhofer, K. J. J. Stability of nanostructured iridium oxide electrocatalysts during oxygen evolution reaction in acidic environment. *Electrochemistry Communications* **2014**, *48*, 81-85. DOI: 10.1016/j.elecom.2014.08.027.
(12) Fleischmann, S.; Mitchell, J. B.; Wang, R.; Zhan, C.; Jiang, D. E.; Presser, V.; Augustyn, V. Pseudocapacitance: From Fundamental Understanding to High Power Energy Storage Materials. *Chem Rev* **2020**, *120*, 6738-6782. DOI: 10.1021/acs.chemrev.0c00170.
(13) Jovanovič, P.; Hodnik, N.; Ruiz-Zepeda, F.; Arčon, I.; Jozinović, B.; Zorko, M.; Bele, M.; Šala, M.; Šelih, V. S.; Hočevar, S.; et al. Electrochemical Dissolution of Iridium and Iridium Oxide Particles in Acidic Media: Transmission Electron Microscopy, Electrochemical Flow Cell

Coupled to Inductively Coupled Plasma Mass Spectrometry, and X-ray Absorption Spectroscopy Study. *J. Am. Chem. Soc.* **2017**, *139*, 12837–12846. DOI: 10.1021/jacs.7b08071.
(14) Natarajan, K.; Munirathinam, E.; Yang, T. C. K. Operando Investigation of Structural and Chemical Origin of Co3O4 Stability in Acid under Oxygen Evolution Reaction. *ACS Applied Materials & Interfaces* **2021**, *13*, 27140-27148. DOI: 10.1021/acsami.1c07267.
